# Pleuropulmonary Kaposi Sarcoma in the Setting of Immune Reactivation

**DOI:** 10.4172/2161-105X.1000352

**Published:** 2016-05-30

**Authors:** Karthik Suresh, Roy Semaan, Sixto Arias, Petros Karakousis, Hans Lee

**Affiliations:** 1 Department of Medicine, Division of Pulmonary and Critical Care medicine, Johns Hopkins School of Medicine, Baltimore, USA; 2 Department of Medicine, Division of Infectious Diseases, Johns Hopkins School of Medicine, Baltimore, USA

**Keywords:** Kaposi sarcoma, IRIS, Pleural effusion, Malignant pleural effusion, Thoracoscopy

## Abstract

We present a case of a 26 year with history of HIV/AIDS who presented with a pleural effusion. Serial radiography, pleural fluid analysis as well as clinical symptoms revealed development of Kaposi Sarcoma related immune reconstitution inflammatory syndrome (KS-IRIS) in the setting of initiation of effective anti- retroviral therapy.

## Introduction

R.S. is a 26 year-old with HIV/AIDS, diagnosed in 2008, with intermittent compliance with antiretroviral therapy (Darunavir/ Truvada/Ritonavir), who presented to our hospital in 2014 with complaints of headache and vision changes, and was diagnosed with syphilis. During the admission, violaceous papules were noted on his skin, and a biopsy revealed histologic findings characteristic of Kaposi Sarcoma (KS), including proliferation of spindle cells, prominent, slit-like vascular spaces, and extravasated red blood cells. His viral genotype showed mutations in the reverse transcriptase (A62V, K65R, L100I, K103N, M184I, P225H) and protease (A71T) genes, indicating resistance to lamuvidine, emtricitabine, abacavir, efavirenz and nevirapine. His regimen was switched to Dolutegravir, Ritonavir, Darunavir and Rilpivirine. He had no pulmonary complaints at that time.

**Figure 1:**
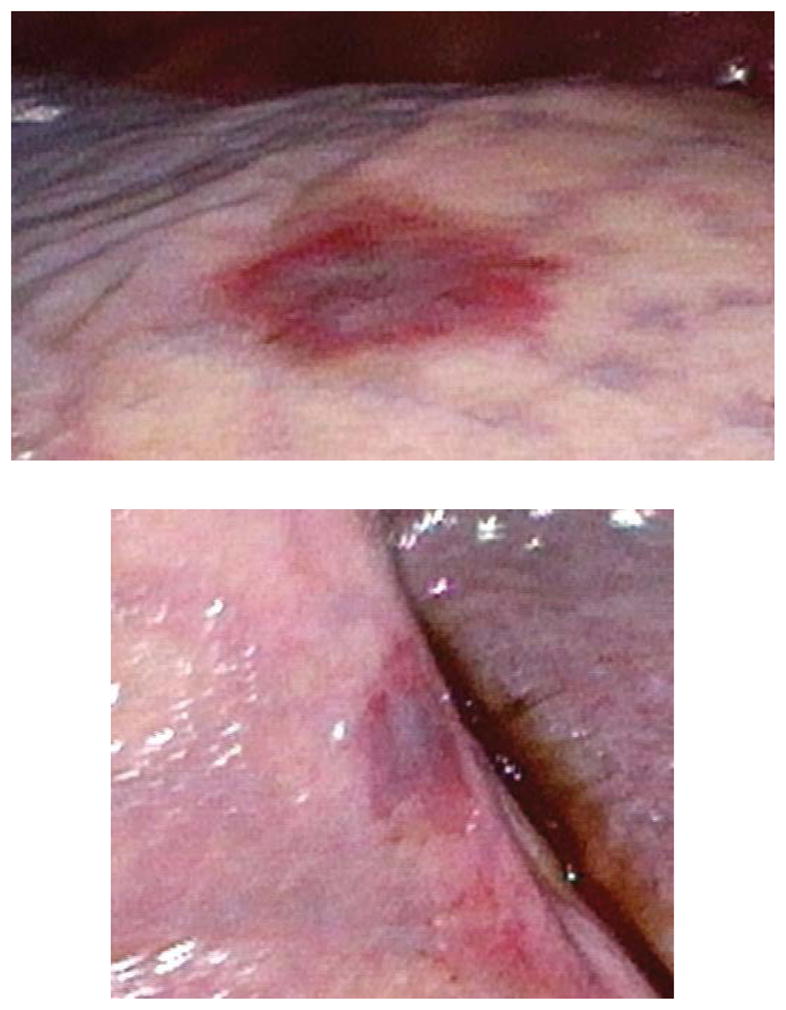
Pleural KS lesions.

One month later, he was admitted with an upper extremity deep venous thrombosis and found to have compression of his right axillary vein from lymphadenopathy. He continued to have no pulmonary symptoms, but a right pleural effusion was noted on imaging. A thoracentesis showed lymphocyte predominant (247 cells/mL, 87% lymphocytes) with pleural/serum albumin ratio of 0.64. Microbiologic cultures were negative; flow cytometric and cytologic analyses were not done. One month after this admission, he presented with complaints of dyspnea and was found to have an enlarging right-sided pleural effusion. Of note, he reported strict medical adherence to his new ARV (anti-retroviral) regimen, reflected by a rapid decline in viral load from 72,000 virions/mL to undetectable. Examination was notable for palpable cervical adenopathy and diminished breath sounds on the right side, dullness to percussion and 1–3 cm long raised violaceous nodule on the inner aspect of left upper extremity. Thoracentesis yielded 1400 cc of serosanguinous fluid that was exudative (pleural/serum albumin ratio of 0.77) and lymphocyte- predominant (946 cells/mL, 91% lymphocytes). Microbiological, fungal and mycobacterial cultures were all negative. Cytology showed reactive mesothelial cells and flow cytometry was negative for malignancy. Following drainage, the pleural effusion rapidly re- accumulated and a thoracoscopy was performed, which showed characteristic KS lesions in the visceral pleura (Figure 1). Talc pleurodesis was performed at the end of thoracoscopy. Cultures for aerobic, anaerobic fungus and mycobacteria were negative. A few weeks later, he was started on chemotherapy for pleuropulmonary KS.

On follow-up, no recurrence of the pleural effusion was noted.

## Discussion

Kaposi Sarcoma Herpes Virus (KSHV, or HHV-8) is a double-stranded DNA virus that is associated with several clinical entities in both immunodeficient and immunocompetent patients: Castleman’s disease, Primary Effusion Lymphoma and, as in our case, Kaposi’s Sarcoma, depending on the cell type infected by KS virus. KS often presents with dermatologic manifestations but can metastasize to involve the lungs, liver and lymph nodes. Pleural and/or pulmonary manifestations occur in about half of KS patients [1]. Pulmonary manifestations include cough, dyspnea, hemoptysis, parenchymal nodular lesions, adenopathy and pleural effusions. Notably, about 15% of the patients with pulmonary KS have no evidence of mucocutaneous KS [2]. Most pathognomonic, however, is the presence of endobronchial lesions; visualization of these lesions, which often occur at vessel branch points, is usually sufficient for presumptive diagnosis. The pleural lesions of KS appear as visceral red to violaceous plaques and are typically unilateral with an associated effusion that is serosanguineous, exudative and mononuclear. Histologically, KS is a mesenchymal malignancy that compromises blood and lymphatic vessels [3]. A fully developed lesion consists of interwoven bands of spindle cells and vascular structures embedded in a network of reticular and collagen fibres [4]. HAART (Highly Active Anti-Retroviral Therapy) is the cornerstone therapy, but can be complicated by the development of Immune Reconstitution Inflammatory Syndrome (IRIS).

IRIS is a complication to initiation of ART (anti-retroviral therapy) that is fairly uncommon in western countries [5]. It is characterized by a robust response to anti-retroviral therapy (usually with rapid decline in viral load, as seen in our patient) and clinical deterioration of a clinical condition with an infectious causative agent (such as KS) [6,7]. Prior to initiation of effective antiviral therapy, components of the HIV viral machinery (such as transactivating protein, or *Tat)* promote KSHV survival and tumorigenesis within infected cells, leading to continued infection and potential malignant transformation [8,9]. Additionally, patients with HIV and progressive KS may also have diminished T cell responses to KSHV antigens [10]. Our patient began experiencing symptoms of both cutaneous and pleural KS prior to initiation of effective ART. When HAART therapy is initiated and T-cell responses are restored, an exuberant reaction to resident KSHV may occur, thus leading to clinical progression of KS [11]. Serial imaging and lab work in our patient showed worsening pleural effusions following initiation of effective ARV and concomitant drop in viral load (Table 1). As serum absolute lymphocyte count (ALC) recovers, there is an increase in pleural lymphocytes and clinical worsening of pleural effusions suggestive of increased inflammation (Table 1). This timeframe of clinical deterioration (6–8 weeks) following ART initiation is consistent with prior reports of KS-related immune reconstitution inflammatory syndrome (KS- IRIS [12]). Prior case series examining radiographic findings in KS-IRIS [13] have reported findings similar to ours, including increased pleural effusions associated with development and worsening of KS-IRIS [13]. However, in our patient, the symptomatic nature of the effusions necessitated multiple thoracenteses, which enabled us to characterize the natural history of KS-IRIS associated pleural effusions through serial pleural fluid analyses. The timing of HAART in the setting of KS-IRIS remains unclear, but evidence from study of TB and HIV suggests that HAART should not be delayed out of concern for opportunistic infection related IRIS [14]. Indeed, in our patient, with continued HAART therapy as well as local interventions (pleurodesis), the effusions resolved.

## Conclusions

In summary, we present a case of pleuropulmonary KS with clinical deterioration following ART initiation consistent with KS-IRIS. The diagnosis in the case was made via thoracoscopy, but further insight into the pathophysiology of the natural history of KS and IRIS was gained through serial analysis of pleural fluid.

## Figures and Tables

**Table 1:** Time course of pleural fluid changes. VL: Viral Load; ALC: Absolute Lymphocyte Count; WBC: White Blood Cell Count; (serum); KS: Kaposi Sarcoma.

	Initial presentation	1 mo. later	2 mo. later	3 mo. later
CD4 (cells/ mm^3^)	5	22	-	50
VL (copies/mL)	72,000	Undetectable	-	-
ALC (cells/mm^3^)	460	530	780	430
WBC (cells/mm^3^)	1.32	1.19	1.29	2.6
Pleural Lymphocytes (cells/mm^3^)		230	870	-
Pleural total cells		247	946	-
Notes	Admitted with neurosyphilis, switched to new ART regimen due to resistance	Admitted with upper extremity edema, found to have skin KS and lymph node biopsy positive for KS	Thoracoscopy showed pleural KS lesions. Pleurodesis performed during thoracoscopy	-
